# Does YouTube™ Provide Adequate Information on Oral Health During Pregnancy?

**DOI:** 10.7759/cureus.57887

**Published:** 2024-04-09

**Authors:** Şeyma Çardakcı Bahar, Savaş Özarslantürk, Erkan Özcan

**Affiliations:** 1 Periodontics, Gulhane Faculty of Dental Medicine, University of Health Sciences, Ankara, TUR; 2 Dentomaxillofacial Radiology, Gulhane Faculty of Dental Medicine, University of Health Sciences, Ankara, TUR

**Keywords:** youtube, pregnancy, oral health, gingivitis, e-health

## Abstract

Background

This study aimed to assess the reliability, quality, and content of the information provided by YouTube™ videos on oral health during pregnancy to reveal the effectiveness of the videos for patients.

Methodology

This cross-sectional study was conducted by two experienced dental specialists. They initiated the study by searching for YouTube™ videos using the keyword 'pregnancy oral health'. The videos were then assessed based on various parameters, including origin, type, number of days since upload, duration, number of views, number of likes and dislikes, and number of comments. The specialists also calculated the interaction index and viewing rate. The reliability and quality of the videos were evaluated using the global quality scale (GQS) and modified DISCERN (mDISCERN) scales, while the content was assessed with the comprehensiveness tailor-made index. The data were analyzed with the Shapiro-Wilk, the Kruskal-Wallis, the post-hoc Bonferroni, and Fisher's exact tests. The significance level was set at P < 0.05.

Results

After reviewing initially 224 videos, 129 were included in the study. Health professionals were the publishers of most videos. A statistically significant positive correlation was found between content scores and video duration, number of comments, interaction index, and total DISCERN scores (p<0.05) (r=0.445, r=0.186, r=0.552, r=0.241, r=0.200, r=0.681, respectively). Statistically significant associations were found between GQS scores, video duration, number of comments, and total mDISCERN scores (p<0.05) (r=0.510, r=225, r=0.156, r=0.768, respectively). Statistically significant relationships were identified between the total content score, video source, and GQS (p<0.05). According to the total content score, 57.4% of the videos had a score of 2, 35.7% had a score of 1, and only 7% had a score of 0.

Conclusions

This study's findings underscore the significant variability in the scientific accuracy, content, and quality of health information on the Internet, particularly on YouTube™. It reveals that, while there are videos that provide rich content and high-quality information, there are also poor-quality and inadequate videos that may mislead patients. Health professionals should be aware of misinformation found on YouTube™ and ensure that patients always have access to accurate and reliable information.

## Introduction

Pregnancy is a physiological process characterized by changes in a woman's hormone levels, immune system, and metabolism. During this period, altered progesterone and estrogen levels render the host more susceptible to oral infections due to decreased immunity [1]. Elevated estrogen levels have been associated with conditions such as gingivitis, gingival hyperplasia, dental caries, pyogenic granulomas, dry mouth, and dental erosion [2]. The oral health of a pregnant woman may have implications for both her own well-being and that of her unborn child. Inadequate oral hygiene in the mother has been identified as a contributing factor to conditions such as preterm delivery, low birth weight, and preeclampsia [3,4].

The prevalence of women who are aware of oral health's importance during pregnancy varies depending on the population and the region studied. However, studies in the literature have revealed that a large proportion of pregnant women have insufficient awareness of oral health and hygiene and lack basic knowledge about oral health [5,6]. Reasons for this include changes in the socioeconomic status and education levels of pregnant individuals, neglect of dental visits during pregnancy, and lack of knowledge about whether it is safe to receive dental treatment during pregnancy [6]. Therefore, the importance of oral health and hygiene during pregnancy needs to be emphasized, and awareness-raising activities should be encouraged. A critical step is to provide more comprehensive access to information and resources on oral and dental health during pregnancy and to ensure that they receive the necessary information and support from health professionals. Awareness initiatives and campaigns are being conducted internationally for this purpose [7,8].

In recent years, social media has also emerged as a popular source of health information for pregnant women. Expectant mothers turn to social media platforms to verify information provided by health professionals and social networks, seek social support, and obtain information. When it is difficult to schedule appointments with healthcare providers, social media is a convenient option to access medical information owing to its anonymity and easy access features [9]. A study involving healthcare professionals and patients revealed that visual elements on social media platforms are more engaging than textual content and aid in comprehending complex information [10]. YouTube™, the second most utilized social media platform globally, offers users access to many medical topics. However, due to its open upload policy, the information disseminated on YouTube can often be inaccurate and misleading [11]. Such misinformation may lead to confusion and anxiety among pregnant individuals, who are undergoing a unique phase in their lives [12].

In light of these considerations, our study aims to evaluate the content, quality, and reliability of YouTube™ videos on oral health in pregnancy and to assess their adequacy in providing comprehensive information on the topic. Although there are many studies on YouTube content in the literature, there are no studies on the oral dental health of pregnant women.

## Materials and methods

Study design and search strategy

**Figure 1:**
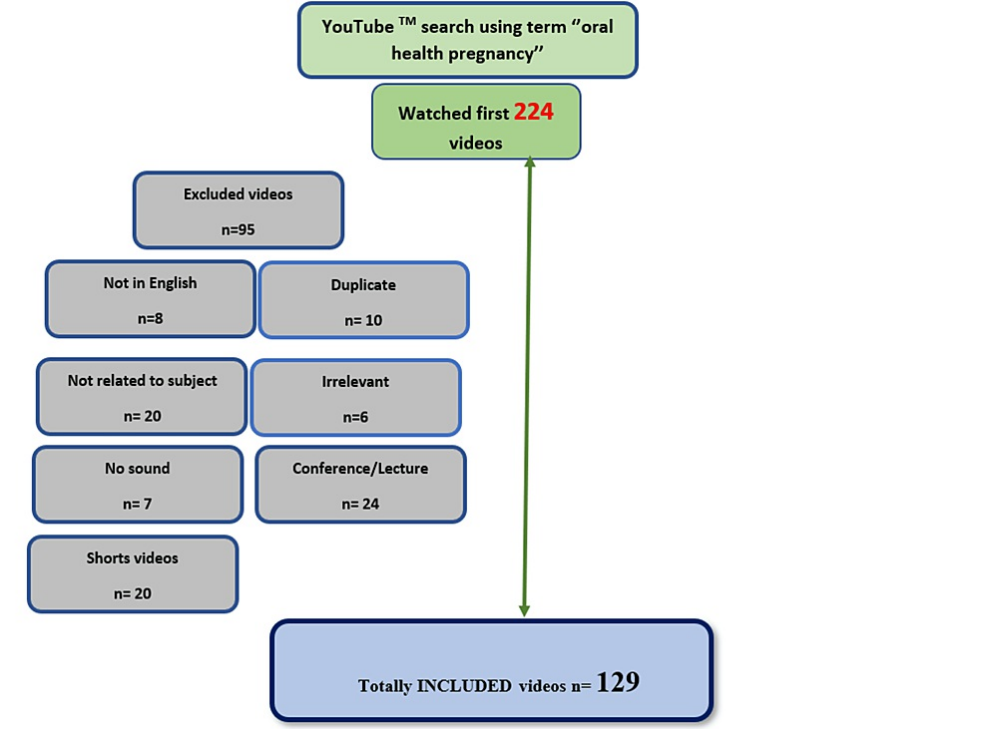
Flow diagram of the selection process

This cross-sectional study aimed to assess the educational value of YouTube™ videos. Ethical approval was not sought since only publicly available data were utilized. Using the Google Trends website (Mountain View, CA), the search terms 'pregnancy oral health', 'gestation oral health', oral health during pregnancy,' 'pregnancy oral care', and 'dental health pregnancy' were compared worldwide over the past five years as of January 27, 2024. The term 'pregnancy oral health' was chosen as the search term for this study (Figure 1). A new email address was created for a YouTube™ account. No filters were applied for YouTube™ video search, and the default sorting option was set to 'related.' The term 'incognito mode' was activated to prevent prior searches from influencing new results. The study population comprised videos on YouTube™ about oral and dental health during pregnancy, limited to 224 videos. Studies indicate that YouTube™ users typically utilize the first three pages, or approximately 30 videos, to gather information [13].

Selection of the videos

English-language videos addressing oral health during pregnancy were included in the study. Irrelevant, non-English, duplicate, conference or lecture videos, videos without sound, advertisements, and short videos were excluded. Before the comparison, computer history and cookies were cleared to prevent any biases from user history. The initial video evaluation was conducted by S.Ö, followed by independent assessments by two specialist dentists, S.Ö and E.Ö, trained in gingivitis, periodontitis, pyogenic granuloma, dental caries, and dental abrasions during pregnancy. The evaluation occurred on January 27-28, 2024, between 07:00 a.m. and 09:00 p.m. For each video, duration, number of views, number of likes/dislikes, country information, number of comments, and upload date were recorded [14]. To prevent bias, researchers were blinded to the number of comments, likes/dislikes, and views until completing their review. Viewer interactions were calculated based on the viewing rate (number of views/number of days since upload date x 100%) and interaction index (number of likes - number of dislikes / total number of views * 100%) [15]. The videos included in the study were cataloged in an Excel file with uniform resource locators (URLs) and video titles as a backup.

Analysis of the videos

According to the inclusion and exclusion criteria, 129 videos were included in the study, while 95 videos were excluded (Figure 1). The source of the videos was categorized as health professionals (dentists, periodontologists), university/hospital/dental clinics, laypersons, commercial entities (dental supply companies or dental manufacturing companies), and others (TV channels, news agencies). Videos were further classified into two types: educational and experiential [16]. Disagreements between the investigators about some videos during the evaluation of the videos were resolved by discussing the issues, reviewing the literature, and consulting with the third expert physician in the study (Ş.Ç.B).

Video quality evaluation indexes

Global Quality Scale (GQS)

The GQS, assessed on a five-point Likert scale, was employed to gauge the quality of the videos included in the study, their utility for patients, and the accessibility of information [17]. According to this scale, videos are scored as follows: 1 for very poor quality, 2 for generally poor with limited details, 3 for moderate quality, 4 for good quality, and 5 for excellent quality.

Modified DISCERN Scoring System

The reliability and quality of the videos were evaluated using the modified-DISCERN (mDISCERN) scale. The DISCERN scale is designed to assess the quality of information regarding treatment options for specific health issues (http://www.discern.org.uk). The mDISCERN scale evaluates visual information and media [13]. It comprises five questions, each rated on a five-point Likert scale. A score of 0 is assigned for a "no" answer and 1 point for a "yes" answer for each question. Higher scores indicate more excellent quality and reliability of the videos.

Comprehensiveness Tailor-Made Index (Total Content Score)

A customized comprehensiveness index was utilized to assess the content of the videos based on clinical signs, risk factors (etiological factors), treatment options, and the readability of the information provided. Each video received a score on a 0-2 point scale [18]. A score of 0 was assigned if the video did not mention any risk factor, clinical condition, or treatment. A score of 1 was given if the video mentioned at least one etiological factor and one clinical condition but did not provide information on the treatment. A score of 2 was awarded if the video covered at least two etiological factors, and at least two clinical conditions, and included information on treating at least one condition (Table 1). 

**Table 1:** Comprehensive tailor-made index (total content score) Score 0: The video did not describe any clinical presentations, risk factors, or management options. Score 1: The video described one clinical presentation and one aetiological factor and did not describe management options. Score 2: The video described at least two clinical presentations, at least two aetiological factors, and at least one management option.

Clinical Presentation	Etiological Factors	Management
Gingival Inflammation	Hormonal changes, increased blood flow	Good oral hygiene, regular dental visits
Gingival Hyperplasia	Hormonal changes, increased blood flow	Good oral hygiene, professional dental cleaning
Dental Caries	Increased carbohydrate intake, hormonal changes	Regular brushing with fluoride toothpaste, balanced diet
Pregnancy Tumors (Pyogenic Granulomas)	Hormonal changes, local irritants	Professional removal if necessary
Xerostomia	Hormonal changes, dehydration	Increased fluid intake, sugar-free gum, saliva substitutes
Tooth Mobility	Hormonal changes, ligament laxity	Monitoring, possibly stabilization with splints
Increased Susceptibility to Periodontal Disease	Hormonal changes, increased acid production	Regular dental check-ups, dietary advice
Tooth Erosion Due to Vomiting	Frequent vomiting, gastric acid exposure	Rinsing mouth with water after vomiting, fluoride remineralization, dental evaluation
Dental Caries	Increased carbohydrate intake, hormonal changes	Regular brushing with fluoride toothpaste, balanced diet

Statistical analysis

Descriptive statistics, including number, percentage, mean, standard deviation, median, minimum, and maximum, were presented in this study. Normality assumption was assessed using the Shapiro-Wilk test. The Kruskal-Wallis test was employed to compare the means of three or more groups with non-normal distribution. The post-hoc Bonferroni test was conducted to identify the group or groups responsible for any observed differences. Kendall's Tau correlation was used to explore the relationship between continuous and categorical variables. Spearman's correlation coefficient was calculated to examine the relationship between two continuous variables unsuitable for normal distribution. Fisher's exact test was utilized to investigate the relationship between categorical variables when the sample size assumption (expected value > 5) was not met. Statistical analysis was performed using IBM SPSS Statistics version 25.0 for Windows (IBM Corp., Armonk, NY). The significance level was set at p < 0.05.

## Results

This study evaluated 124 videos. Table 2 illustrates the distribution of videos included in the study according to the countries where they were uploaded, while Table 3 and Table 4 present the features and characteristics of the included YouTube™ videos. According to the analysis results, a statistically significant difference was observed in the averages of video duration, number of comments, Interaction index, and total DISCERN scores based on the total content scores (p < 0.05). Specifically, for 'video duration,' statistically significant differences were found between score 2 and scores 0 and 1 (p < 0.001, p < 0.001 ), with the average of score 2 being higher than those of scores 0 and 1. For total DISCERN, statistically significant differences were noted between score 2 and scores 0 and 1 (p < 0.001, p < 0.001 ), with the average of score 2 being higher (Table 5). Statistically significant relationships were identified between the total content score, video source, and GQS (p < 0.05). Specifically, when the video source was health professionals, the total content score was predominantly 2. Videos with a GQS of 2 generally had a total content score of 1, while those with a GQS of 3, 4, and 5 predominantly had a total content score of 2. Upon analyzing the responses to DISCERN questions, it was observed that videos receiving a 'yes' answer tended to have a total content score of 2, while those with a 'no' answer tended to have a total content score of 1. Additionally, as the total DISCERN scores increased, the total content score also tended to increase (Table 6).

**Table 2:** Distribution of countries where the videos were uploaded

Countries	n	%
USA	85	65.8
Australia	7	5.5
United Arab Emirates	2	1.6
Bangladesh	1	0.8
South Africa	1	0.8
India	21	16.3
Ireland	1	0.8
Canada	1	0.8
Malaysia	1	0.8
Saudi Arabia	1	0.8
UK	7	5.4

**Table 3:** YouTube video features

	Minimum	Maximum	Mean	Standard Deviation	Median
Number of views	6	385,348	7960.64	35,441.12	700
Duration in minutes	0.25	22.88	3.48	3.46	2.50
Days since upload	35	5480	1858.50	1308.33	1610
Number of comments	0	210	4.29	20.67	0
Number of likes	0	1200	35.10	116.01	5
Viewing rate	0.37	16143.61	392.56	1492.86	45.22
Interaction index	0	35.29	1.58	3.98	0.49
Total DISCERN	0	5	2.71	1.10	3

**Table 4:** YouTube video characteristics

		n	%
Source of Upload	Healthcare professionals	84	65.1
	Hospital/university	14	10.9
	Commercial	8	6.2
	Layperson	9	7.0
	Other	14	10.9
Video Type	Educational	127	98.4
	Testimonial	2	1.6
Total Content Score	Score 0	9	7.0
	Score 1	46	35.7
	Score 2	74	57.4
Global Quality Scale	Score 1	3	2.3
	Score 2	36	28.1
	Score 3	46	35.9
	Score 4	31	24.2
	Score 5	12	9.4

**Table 5:** Distribution and comparison of video features based on the total content scores *p < 0.05 and †: Pearson chi-square test

		Minimum-maximum	Mean±standard deviation (median)	Test statistics	p
Number of views	Score 0	52-20,736	8110.67±7026.28 (9850)	3.544	0.170
	Score 1	7-385,348	12734.85±57,495.25 (489.5)		
	Score 2	6-77,899	4974.64±11,655.58 (886)		
Duration in minutes	Score 0	0.5-1.8	1.1±0.55 (0.93)	39.178	<0.001*
	Score 1	0.25-8.62	2.09±1.61 (1.64)		
	Score 2	0.82-22.88	4.63±4.02 (3.38)		
Days since upload	Score 0	250-3151	1878.56±967.92 (2010)	1.914	0.384
	Score 1	35-5480	1710.09±1423.95 (1451.5)		
	Score 2	79-4377	1948.31±1275.43 (1618.5)		
Number of comments	Score 0	0-5	1.56±2.19 (0)	10.488	0.005*
	Score 1	0-80	2.2±11.86 (0)		
	Score 2	0-210	5.93±25.6 (0)		
Number of likes	Score 0	0-91	28.22±30.63 (30)	3.693	0.158
	Score 1	0-203	17.76±44.63 (2.5)		
	Score 2	0-1200	46.72±148.1 (5)		
Viewing rate	Score 0	8.41-803.35	357.85±290.4 (412.25)	2.261	0.260
	Score 1	0.57-16,143.61	618.68±2418.8 (48.47)		
	Score 2	0.37-2903.43	256.22±490.64 (34.19)		
Interaction index	Score 0	0-0.56	0.24±0.23 (0.28)	7.938	0.019*
	Score 1	0-20	1.11±3.01 (0.32)		
	Score 2	0-35.29	2.04±4.66 (0.68)		
DISCERN	Score 0	1-3	1.44±0.73 (1)	73.017	<0.001*
	Score 1	0-3	1.89±0.74 (2)		
	Score 2	2-5	3.38±0.81 (3)		

**Table 6:** The relationships between video characteristics and total content scores *p < 0.05 †: Pearson chi-square test

		Score 0		Score 1		Score 2			
		n	%	n	%	n	%	Test statistics	p
Source of Upload	Healthcare professionals	4	44.4	23	50.0		77.0	16.284	0.018*
	Hospital/university	3	33.3	6	13.0	5	6.8		
	Commercial	0	0.0	5	10.9	3	4.1		
	Layperson	1	11.1	6	13.0	2	2.7		
	Other	1	11.1	6	13.0	7	9.5		
Video Type	Educational	9	100.0	44	95.7	74	100.0	3.372	0.258
	Testimonial	0	0.0	2	4,3	0	0.0		
Global Quality Scale	Score 1	3	33.3	0	0.0	0	0.0	103.270	<0.001*
	Score 2	5	55.6	30	65.2	1	1.4		
	Score 3	1	11.1	16	34.8	29	39.7		
	Score 4	0	0.0	0	0.0	31	42.5		
	Score 5	0	0.0	0	0,0	12	16.4		
Are the aims clear and achieved?	No	6	66.7	28	60.9	3	4.1	51.589†	<0.001*
	Yes	3	33.3	18	39.1	71	95.9		
Are reliable sources of information used?	No	3	33.3	13	28.3	4	5.4	13.659†	0.001*
	Yes	6	66.7	33	71.7	70	94.6		
Is the information presented balanced and unbiased?	No	6	66.7	9	19.6	0	0.0	39.089†	<0.001*
	Yes	3	33.3	37	80.4	74	100.0		
Are additional sources of information listed for patient reference?	No	8	88.9	46	100.0	53	71.6	16.391†	<0.001*
	Yes	1	11.1	0	0,0	21	28.4		
Are areas of uncertainty mentioned?	No	9	100.0	46	100.0	60	81.1	12.248	0.003*
	Yes	0	0.0	0	0.0	14	18.9		
Total DISCERN	Score 0	0	0.0	2	4.3	0	0.0	90.614	<0.001*
	Score 1	6	66.7	9	19.6	0	0.0		
	Score 2	2	22.2	27	58.7	6	8.1		
	Score 3	1	11.1	8	17.4	43	58.1		
	Score 4	0	0.0	0	0.0	16	21.6		
	Score 5	0	0.0	0	0.0	9	12.2		

A positive, moderate, and statistically significant relationship was found between video duration and total content score, and similar relationships were observed between video duration and GQS, between video duration and total DISCERN, between the interaction score and total content score, between the interaction score and GQS, and between total DISCERN and total content scores (p < 0.05). In addition, a positive, low, and statistically significant relationship was found between the number of comments and total content score, between the number of comments and GQS, between the number of comments and total DISCERN, between the interaction score and total content score, and between the interaction score and GQS (p < 0.05). Finally, a positive, high-level, and statistically significant relationship was found between the total DISCERN score and GQS (p < 0.05) (Table 7).

**Table 7:** Relationships between the characteristics of YouTube videos and total content score, total global scale, and total DISCERN *p < 0.05, r: Correlation coefficient and ⁑: Spearman correlation

		Total Content Score	Total Global Scale	Total DISCERN^ ⁑^
Number of views	r	-0.005	0.081	0.083
	p	0.944	0.225	0.352
Duration in minutes	r	0.445	0.510	0.552
	p	<0.001*	<0.001*	<0.001*
Days since upload	r	0.068	0.118	0.119
	p	0.336	0.077	0.179
Number of comments	r	0.186	0.225	0.241
	p	0.021*	0.003*	0.006*
Number of likes	r	0.091	0.137	0.129
	p	0.212	0.049	0.146
Viewing rate	r	-0.053	0.022	0.019
	p	0.456	0.745	0.833
Interaction index	r	0.200	0.156	0.158
	p	0.006*	0.024*	0.074
Total DISCERN	r	0.681	0.768	1.000
	p	<0.001*	<0.001*	-

## Discussion

With the increasing digitalization and the growing prominence of social media applications in our daily lives, platforms such as YouTube™ have emerged as significant sources of medical information due to their accessibility regardless of time and geographical location. However, the ability for independent users to upload videos raises concerns regarding the accuracy and reliability of content on YouTube™ [19]. One study showed that Internet-based information significantly influenced decision-making processes, and 83% of pregnant women acknowledged this influence [20]. Furthermore, another study reported that pregnant women reported increased health concerns and difficulties in accessing accurate information, especially during events such as the COVID-19 pandemic, which increased their concerns about their unborn child [21]. Therefore, online information should be understandable, informative, and based on scientific evidence. The aim of this study was to evaluate the quality, reliability, and content of YouTube™ videos in order to ensure that healthcare professionals are aware of the information presented in YouTube™ videos and can accurately and adequately inform pregnant patients about their oral health and to ensure that pregnant patients receiving healthcare services use the right resources for their oral health.

There are studies in the literature examining the quality and reliability of YouTube™ videos in medicine and dentistry [22-24]. According to our literature review, this is the first study to analyze videos uploaded on YouTube™ on oral health in pregnancy. The number of views of the 129 videos included in this study ranged from 6 to 385,348. This shows that the viewership of videos can reach high numbers and mislead many people when the content is inadequate. In this study, we used reliability scores adapted from mDISCERN and GQS to measure the overall quality and reliability of the videos, similar to previous YouTube™ studies [23-25]. DISCERN plays an important role in enabling patients to make informed treatment choices based on accurate evidence by raising the standards of consumer health information [13,17]. In this respect, we believe that the reliability scores adapted from DISCERN and GQS are sufficient for video evaluation.

Previous studies have reported that 75% of videos on oral leukoplakia were created by independent users and company advertisements, 65% of videos on orthodontic pain were created by laypersons, and 36.7% of videos on dentin hypersensitivity were created by healthcare professionals [23,25,26]. In this study, 65.1% of the included videos were posted by healthcare professionals, and 10.9% were posted by hospitals and universities. In studies where most video sources were layperson or independent individuals with commercial purposes, very few videos were found to be content-rich, reliable, and useful [25,26]. In this study, most of the videos were created by healthcare professionals, and the videos received moderate-content (35.7%) or high-content (57.4%) scores.

In this study, video duration, number of comments, interaction index, total DISCERN scores, and GQS scores of videos with rich content were found to be higher than videos with poor content. Topsakal et al. also found that the reliability score, average length, and GQS of videos with rich content were statistically significantly higher than videos with poor content [27]. Albayrak et al. also found that GQS score, DISCERN, video duration, and number of comments were higher in the rich content video group compared to the poor content video group [28]. This explains the longer duration of rich-content videos because they mention more content.

In this study, a positive significant correlation was found between video duration, number of comments, interaction index and total DISCERN scores, total content scores, and total DISCERN scores and GQS values. Saraç Atagün et al. stated that quality and reliable videos with rich content have a longer duration and receive more comments [23]. Since these videos are more informative, it is expected that they will receive more comments and have a higher interaction index. On the other hand, Sadry et al. found in their study that the attention of the viewers decreased due to the long duration of good and high-quality videos and that this situation had a viewership rate. In this study, no significant difference was found between the duration of the videos and the viewership rate. The reason for this could be the variability in platform algorithms or user behaviors, leading to differences in the preference for short or long videos depending on the type of content viewers prefer [29].

This study also found that oral health videos' reliability, quality, and comprehensiveness during pregnancy on YouTube™ were significantly positively associated with duration, number of comments, and interaction index but not with view rate, number of likes, and number of views. Ceylan Şen et al. also reported in their oral candidiasis study that videos' reliability, quality, and comprehensiveness are not always associated with popularity and number of viewers. Individuals who watch videos do not question and evaluate quality and content [30].

It is important to focus on increasing access to accurate and reliable information on oral health during pregnancy. Developing educational materials such as brochures, online resources, and mobile apps specifically designed to address the oral health needs of pregnant individuals; integrating oral health education into prenatal care visits; and creating engaging and informative content through social media and digital platforms such as YouTube™, in collaboration with trusted influencers and health professionals, can help reach a wider audience and address common misconceptions or concerns about oral health during pregnancy.

Limitations

Our study has some limitations. In the absence of a validated evaluation tool for video-based resources, video evaluations were conducted by two independent researchers according to our own checklist. A second limitation is that YouTube™ search results are dynamic. Results may change when new videos are uploaded, old videos are deleted, or the search word is changed. Therefore, this cross-sectional study shows the usefulness of videos on oral health in pregnancy when the videos were downloaded. Finally, cross-sectional studies examining English language videos on YouTube™ videos on the topic in the local language of the countries were excluded.

## Conclusions

YouTube™ is an open-access and popular video-sharing platform that hosts a large number of videos in the healthcare domain. According to this study, videos uploaded by healthcare professionals have medium-to-high content scores but also include videos with low content and poor quality. Health professionals should warn pregnant women about viewing unreliable content, as viewing unreliable content can have negative consequences. In addition, no significant relationship was found between content score, GQS, total DISCERN scores, and the number of views in this study. Therefore, the content and quality of popular videos on oral health during pregnancy should be improved. In the field of health information, users should be directed to high-quality and reliable videos uploaded by health professionals. By emphasizing the importance of oral health during pregnancy, this study aims to encourage healthcare professionals to produce more informative and content-rich videos. With this approach, we believe that digital platforms such as YouTube™ can be utilized to disseminate accurate information and raise awareness.

## Appendices

Global Quality Scale (GQS)

Score 1: Poor quality, poor flow of the video, most information missing, not at all useful for patients

Score 2: Generally poor quality and flow, some information listed but many important topics missing, of very limited use to patients

Score 3: Moderate quality, suboptimal flow, some important information adequately discussed but others poorly discussed, somewhat useful for patients

Score 4: Good quality and generally good flow. Most of the relevant information is listed but some topics are not listed. Useful for patients

Score 5: Excellent quality and flow, very useful for patients

Reliability of Information (MDISCERN) (1 Point for Every Yes, 0 Points for No)

1. Are the aims clear and achieved?

2. Are reliable sources of information used?

3. Is the information presented balanced and unbiased?

4. Are additional sources of information listed for patient reference?

5. Are areas of uncertainty mentioned?
